# Aqueous Extracts of* Herba Cistanche* Promoted Intestinal Motility in Loperamide-Induced Constipation Rats by Ameliorating the Interstitial Cells of Cajal

**DOI:** 10.1155/2017/6236904

**Published:** 2017-12-28

**Authors:** Shuai Yan, Yin-zi Yue, Xiao-peng Wang, Hong-li Dong, Shu-guang Zhen, Ben-sheng Wu, Hai-hua Qian

**Affiliations:** ^1^Suzhou Hospital of Traditional Chinese Medicine, Suzhou, Jiangsu 215009, China; ^2^Nanjing University of Traditional Chinese Medicine, Nanjing, Jiangsu 210023, China; ^3^Department of Anorectal Surgery, Jiangsu Provincial Hospital of Traditional Chinese Medicine, Nanjing, Jiangsu 210029, China

## Abstract

Traditional Chinese medicine was reported to have good effects in treating functional constipation. This work attempted to prove the effects of aqueous extracts of* Herba Cistanche* (AEHC) on STC treatment and to determine the possible mechanisms by a loperamide-induced slow transit constipation (STC) model. HPLC was performed for identification and confirmation of the bioactive components in the AEHC. It was found that AEHC attenuated STC responses based on increased fecal quantity, moisture content, and intestinal transit rate, as well as serum levels of GAS, MTL, SS, and CGRP. The protein and mRNA levels of c-kit, a labeling of interstitial cells of Cajal (ICC), also increased. Meanwhile, only the protein level of SCF, a ligand of c-kit, increased. The analysis of our data suggested that AEHC could obviously improve the function of ICC via a signaling pathway involving PI3K, SCF, and c-kit and enhance colonic motility indices such as GAS, MTL, SS, and CGRP. It is interesting to note that AEHC appeared to be effective on constipation, so further experiments are necessary to clarify the exact mechanisms involved.

## 1. Introduction

Constipation is a common functional gastrointestinal disease and public health problem, characterized by continuously difficult, infrequent, or incomplete defecation [1]. The prevalence of constipation varies from 0.7% to 81% around the world, especially in the elderly [2, 3]. Slow transit constipation (STC) is a common gastrointestinal complaint among 13–37% of patients with chronic treatment-resistant constipation [4, 5]. Epidemiological data indicate a higher incidence of STC among young females than males [6]. STC causes intractable constipation, showing little response to conservative treatment and a tendency of neurodegenerative disorder. Not as simple as a functional disease, it calls for invasive and aggressive treatment [7].

A number of current chemical drugs such as osmotic or secretory laxatives and bulking agents are universally used to treat constipation [8], although their applications are limited owing to high expenditures and abdominal side effects such as pain, cramps, and bloating [9]. At present, regulation of the gastrointestinal tract is the primary focus of constipation therapies. Cisapride was first generated as a promotility agent for the treatment of gastric disease but was later withdrawn because of the risk of cardiac arrhythmias [10].

Tegaserod is a selective 5-hydroxytryptamine receptor antagonist that can stimulate intestinal peristalsis and secretion [11, 12]. Prucalopride is a novel dihydrobenzofuran carboxamide derivative and 5-HT_4_ receptor agonist with high selectivity and specificity, which can enhance the gastric, small intestine and colon motility and works faster in dynamics [13–15]. As a prostaglandin E1 derivative, lubiprostone can selectively activate the CIC_−2_ channel to promote gastrointestinal secretion and increase the effects of intestinal transmission [16].

Motility in constipation is a widespread problem, but its etiology is not yet clear. Nevertheless, convincing evidence has demonstrated the contribution of interstitial cells of Cajal (ICC) to pathogenesis of constipation [17]. ICC is found between the nerve endings and smooth muscle cells in the gastrointestinal tract. The ICC are generally acknowledged as pacemaker cells for gastrointestinal activity and as neuromuscular transmission mediators [18]. Studies have indicated that ICC density in the colon of patients with slow transit constipation is remarkably lower compared with those in normal patients [19]. Accordingly, a declining number of ICC may lead to the shortage of slow wave activity, thus affecting the contractile response and inducing delayed transit in slow transit constipation patients. For this point, melioration of ICC using pharmaceuticals may be crucial to cure slow transit constipation.

Various herbal medicines and traditional Chinese formula have recently drawn attention as new-fangled therapeutics for the treatment of chronic constipation as a result of improved stool production [20, 21].* Herba Cistanche* is a traditional herbal medicine used in the treatment of chronic renal disease, female infertility, profuse menorrhagia, and impotence [22, 23].* Herba Cistanche* prevents brain neuron apoptosis by expressing apoptosis-related factors and neurotrophic factors in MES23.5 cells [24].* Herba Cistanche* has also demonstrated pharmaceutical potential in the treatment of Yang-Qi Kidney-Yang Deficiency Syndrome. A recent study has demonstrated that* Herba Cistanche* heightens the mitochondrial respiration and glutathione antioxidant status in H9c2 cardiomyocytes [25].

TCM believes that constipation has a close relationship with kidney. Opening at external genitalia and anus, kidney governs urination and defecation. Transportation of the waste relies on the promotion of kidney qi and nourishment of kidney yin. If kidney yang is deficient or the fire of life gate dies out, cold congestion of large intestine will result in stagnation of waste. Constipation is also caused by dry bowel movement, since deficiency of kidney essence fails to generate enough fluid [26].

*Herba Cistanche* is a common tonifying herb, which is sweet and salty in flavor and warm in nature. Attributive to large intestine and kidney meridians, it can tonify kidney yang and moisten the intestine to relieve constipation. This single herb and its Chinese pharmaceutical preparations have been widely used to treat constipation with remarkable efficacy [27].

In China, the effects of* Herba Cistanche* roots on constipation have been recorded [28, 29] and referred to in Shen Nong's Chinese Materia Medica as the “dried succulent stems of the* Cistanche* species” [30]; however, scientific proof on the effect of* Herba Cistanche* roots on constipation has not existed so far, the clinical effective dose is 20 g per day in Chinese Pharmacopoeia, and the clinical effect dose not increase further with the dosage.

Since* Herba Cistanche* is so scarce [31], standardizing its clinical dosage for the treatment of STC by experimental study not only prevents failed treatment due to inadequate dosage but also keeps patients away from economic loss and even medication misadventure by overuse. We hope the results could lay a scientific foundation of new drugs for constipation. Therefore, it is important to investigate the aqueous* Herba Cistanche's* function of intestinal promotion and its action on ICC in the present study.

## 2. Materials and Methods

### 2.1. Preparation of Aqueous Extract of* Herba Cistanche*

All* Herba Cistanche* were purchased from Nanjing Haichang Chinese Medicine Group Co., Ltd. (Nanjing, China), which were harvested, collected, and processed following standard ethnobotanical practices from the plantations in the Sinkiang area of China. The identity of the plant was ascertained morphologically by Professor Tu Lin Lu of Nanjing University of Chinese Medicine, Nanjing, China. A voucher specimen (NUCMCHS-2015628) was deposited at the College of Health Sciences, Nanjing University of Chinese Medicine, Nanjing, China.

Slices of* Herba Cistanche*, weighing 500 g, were pulverized using an electric blender and extracted in 5000 ml distilled water, after which the aqueous extract was purified at 100°C for 2 h using a circulating extraction equipment (Bilon, China). The extracts were filtered through a 0.22 *μ*m membrane pore (Millipore, Billerica, MA, USA), and the residue obtained from the extracts was dissolved in 4000 ml water. After reflux extraction, the extracts were collected and evaporated to obtain the final sample product. Aqueous extracts were concentrated to dry pellets (1 g/ml) using a rotary evaporator (EYELA, Tokyo, Japan) and stored at −20°C for subsequent usage. Solid samples were reconstituted in distilled water to give the required doses of 10, 20, and 40 mg/kg body weight for the experiment.

### 2.2. Animals

The experimental protocol used in this study was reviewed and approved based on ethical procedures and scientific care by the Suzhou Hospital of Traditional Chinese Medicine-Institutional Animal Care and Use Committee, Suzhou, China (SHTCM-IACUC; approval number: PNU-2016-0003). All male rats (Sprague Dawley strain) with a mean weight of 160 ± 20 g were purchased from Shanghai Laboratory Animal Research Center in accordance with the guidelines of the National Institutes of Health. The animals had free access to a standard diet (AIN-93 M) and tap water ad libitum and were housed individually in stainless-steel wire-mesh cages, well ventilated at 23 ± 1°C, 45 ± 5% relative humidity, and exposed to 12 h natural light and 12 h dark daily.

### 2.3. Experimental Design

The loperamide was used to induce constipation in rats in accordance with previous studies [32, 33]. A total of 60 rats were randomly divided into 5 groups (*n* = 12 per group): A: normal group (NG), B: model group (Lop + vehicle-treated group), C: group of constipation rats treated with low aqueous extract of* Herba Cistanche* (Lop + LAEHC-treated group), D: group of constipation rats treated with medium aqueous extract of* Herba Cistanche* (Lop + MAEHC-treated group), and E: group of constipation rats treated with high aqueous extract of* Herba Cistanche* (Lop + HAEHC-treated group). Group A was treated with normal saline (10 ml/kg); Group B was treated with loperamide (4 mg/kg); Group C was treated with loperamide (4 mg/kg) and low aqueous extract of* Herba Cistanche* (1 g/kg); Group D was treated with loperamide (4 mg/kg) and medium aqueous extract of* Herba Cistanche* (2 g/kg); and Group E was treated with loperamide (4 mg/kg) and high aqueous extract of* Herba Cistanche* (4 g/kg). All rats from normal group were injected with 0.9% sodium chloride, while the others were injected with loperamide in 0.9% sodium chloride twice a day for 3 consecutive days to induce constipation. The aqueous extract of* Herba Cistanche* was suspended in water and was orally administered once daily from day 4 to day 18 in Group C, Group D, and Group E, whereas Groups A and B received a consistent volume of water via gavage.

### 2.4. Chromatographic Condition

HPLC profiling was performed in the Shimadzu Liquid Chromatographic System (Tokyo, Japan), consisting of an LC-20AT pump and a double UV-VIS spectrophotometric detector. The eluents were monitored at 330 nm at 30°C. The isocratic mobile phase consisted of methanol −0.1% formic acid (80 : 20, v/v) and ran at a flow rate of 1.0 ml/min. Chromatographic separation was performed on a Diamonsil C_18_ column (4.6 mm × 250 mm, particle size 5 *μ*m, Beijing Dikma Science and Technology Co., Ltd., Beijing, China).

### 2.5. Analysis of Exercise, Food Intake, Water Intake, Body Weight, and Hair

Alterations in exercise, food intake, water intake, body weight, and hair were observed and recorded daily throughout the experimental period. We observed “alterations in exercise” following the specific steps: the self-made box (60 cm high, 50 cm long, and 80 cm wide with dark peripheral wall) is used for open field test; the white ground was divided into diamonds (12 cm × 12 cm) by black line in dark and quiet room; a 100 W bulb hung 1 m above the center of the box; the rats were placed to the center of the box on the 4th, 6th, 12th, and 18th days of the modeling. The number of squares that rats passed through within 5 minutes was used as the horizontal movement score and the upright time of the rats within 5 minutes was used as the vertical movement score. Horizontal and movement scores plus vertical movement score indicate the amount of exercise of rats. Each rat was measured once [34, 35].

### 2.6. Fecal Parameter Measurement

On the final day of the oral administration, fresh stool pellets from each SD rat were collected into round-bottomed stoppered tubes during 6 h, and the total weights from each group were recorded. To determine the fecal moisture content, we used an electric balance to dry the stool pellets until constant weight is attained and measured the dry weight. The moisture content was calculated by the following equation:

(1) $$\begin{array}{c} \frac{\left(\text{wet weight}-\text{dry weight}\right)}{\text{wet weight}}\times 100\%. \end{array}$$

### 2.7. Analysis of Intestinal Transit Rate

Intestinal transit ratio was conducted according to previously reported protocols [36]; briefly, after 14 days of treatment, all SD rats were subject to 12 h fasting but were allowed free access to water. Gum arable (an aqueous suspension of 20% charcoal and 10% gum arable) was administered orally at a volume of 25 ml/kg of each animal. After 30 min, the animals were humanely sacrificed by cervical dislocation and dissected. The small intestines from the pylorus to the cecum were traversed and the distance covered by the charcoal meal and the total length of the small intestine were measured. For an individual rat, the percent of intestinal transit was calculated as the percentage of distance traveled by the charcoal meal relative to the total length of the small intestine. The following equation calculates the intestinal transit rate (%): distance traveled by the charcoal/distance from pylorus to cecum × 100%.

### 2.8. Blood Sample and Tissue Collection

At the end of the experimental period, rats were subjected to 12 h fasting but were allowed free access to water. All rats were then anesthetized 30 min later by intraperitoneal injection of sodium pentobarbital (50 mg/kg) and placed on a temperature-regulated table. Blood samples were collected and centrifuged at 3500 rpm for 15 min to obtain serum. The colon was split immediately and flushed with normal saline at 4°C and then divided to two pieces. One fragment was fixed in 10% formalin and processed in split paraffin for subsequent immunohistochemical (IHC) analysis, while the other was stored at −80°C until they were assayed.

### 2.9. Assessment of GAS, MTL, SS, and CGRP

The concentrations of gastrin (GAS), motilin (MTL), somatostatin (SS), and calcitonin gene related peptide (CGRP) in the serum were estimated by ELISA using commercially available kits.

### 2.10. Histological Analysis

Transverse colons collected from SD rats were fixed with 10% formalin for 12 h, embedded in paraffin wax, and sectioned into 5 *μ*m thick slices that were stained with hematoxylin and eosin (H&E, Sigma-Aldrich, MO, USA). Morphological features of these sections were observed under light microscopy, after which the villus length, crypt thickness, and muscle thickness were measured using Leica Application Suite (Leica Microsystems, Switzerland).

### 2.11. Immunohistochemical Analysis

We followed the methods of Zhu et al. (2016) [36]; unstained 5 *μ*m sections were cut from paraffin blocks for IHC analysis. The sections were stained with rabbit anti-c-kit (1 : 100) at 4°C overnight. The secondary antibody and avidin-biotin peroxidase complex method were used according to the manufacturer's instructions. An immunoglobulin-negative control was used to eliminate nonspecific binding. Expression levels of c-kit were determined by the semiquantitative method. The tissue sections were then examined using the Carl Zeiss imaging system, and staining intensity was quantified using Image-Pro Plus 6.0 software (Media Cybernetics, Silver Springs, MD).

### 2.12. Real-Time Polymerase Chain Reaction

Gene expression levels of c-kit, SCF, and PI3K mRNAs in the colon were determined using a total RNA extraction kit (TIANGEN Biotech, Beijing, China) according to the manufacturer's protocols. Real-time PCR was performed with ABI StepOnePlus Real-Time PCR System (ABI). SYBR Green I Real-Time PCR Master Mix (QPK201, Toyobo, Japan) was used to detect PCR products. Reactions were performed in triplicate runs. Subsequently, fresh rat colon tissue weighing 0.5 g was homogenized in liquid nitrogen. Total RNA (1 *μ*l) was extracted according to the manufacturer's instructions using the TRIzol Reagent (Invitrogen, USA). A reverse transcription-PCR kit (Takara Biotechnology Co., Inc.) was used to synthesize the first strand of cDNA in accordance with the manufacturer's protocol. All the targeted parameters are shown in Table 1. The cycling protocol was under the following conditions: 95°C for 5 min (DNA denaturation), followed by 40 cycles of 95°C for 15 s, 60°C for 20 s, and 72°C for 40 s. A melting curve analysis was performed at the end of the amplification cycles.

### 2.13. Western Blot Analysis

Protein contents of c-kit and SCF in colon samples homogenates were evaluated by Western blot. In brief, the total proteins of homogenates were determined using a bicinchoninic acid assay (Pierce Biotechnology, Rockford, IL, USA). Sodium dodecyl sulfate-polyacrylamide gel (SDS-PAGE) was used to separate proteins in BioRad electrophoresis system (BioRad Laboratories, Hercules, CA, USA). Proteins were then transferred to polyvinylidene difluoride (PVDF) membranes. After 2 h treatment with blocking buffer in TBS containing 5% nonfat milk at room temperature, PVDF membranes were incubated overnight with 1 : 1000 diluted anti-c-kit (Santa Cruz, CA, USA), 1 : 500 diluted anti-SCF (Sigma, MO, USA), and 1 : 8000 diluted anti-*β*-actin (Kangchen, Shanghai, China) antibodies, respectively. Binding of the primary antibody was detected using the corresponding HRP-conjugated secondary antibody (Beyotime, Jiangsu, China). An enhanced chemiluminescence kit (Pierce Biotechnology Inc., Rockford, IL, USA) and the Odyssey Infrared Imaging System (Gene Company Ltd., Hong Kong) were used for chemiluminescence detection. Housekeeping protein *β*-actin was used as a loading control. The amount of protein expression is presented relative to the levels of *β*-actin.

### 2.14. Statistical Analysis

Measurement data were reported as mean ± standard error of mean (SEM) and analyzed by SPSS 16.0 software (SPSS, Chicago, IL, USA). One-way analysis of variance (ANOVA) followed by Duncan multiple range test was used to determine significant differences in all the parameters. Values were considered statistically significant at *P* < 0.05.

## 3. Results

### 3.1. Composition and Functional Components of* Herba Cistanche*

**Figure 1:**
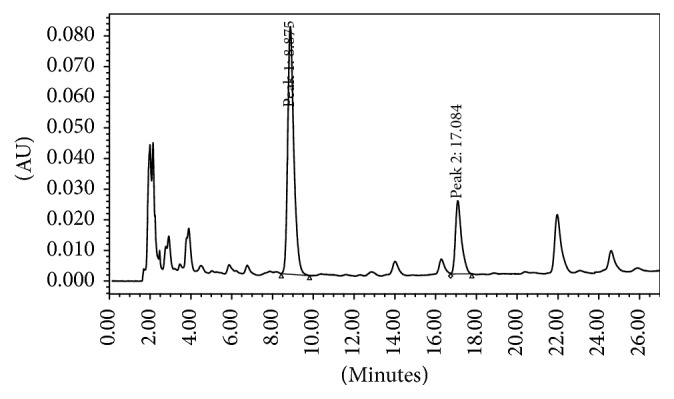
Representative HPLC chromatograms of (Peak 1) echinacoside and (Peak 2) verbascoside.

As shown in Figure 1, the roots of* Herba Cistanche* contain high concentrations of several bioactive components related to laxative effects. The concentrations of total echinacosides and total verbacosides were 0.64 mg/g and 0.16 mg/g, respectively. Two peaks indicating high echinacoside and verbascoside levels were detected in the LP at an appropriate retention time by high-performance liquid chromatography (HPLC).

### 3.2. General Observation

**Figure 2:**
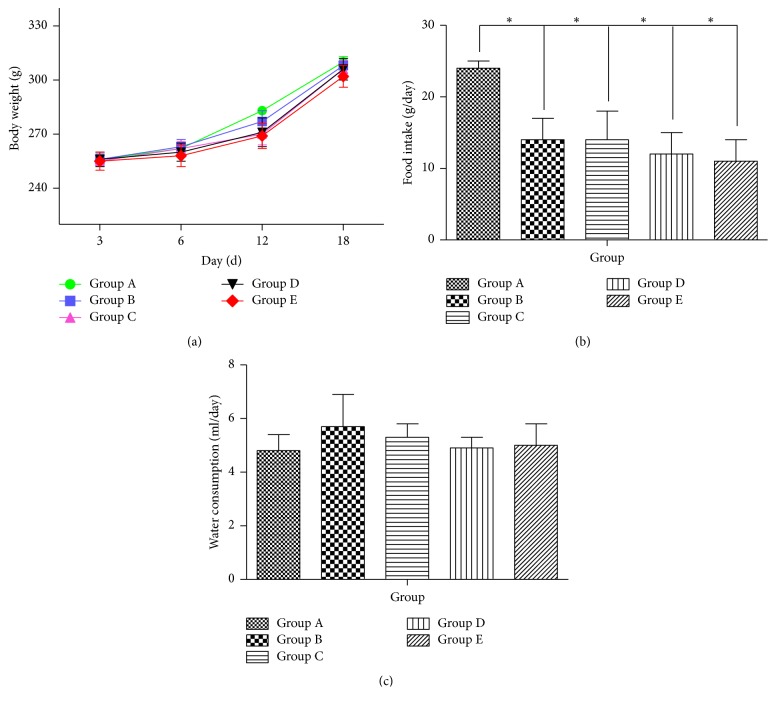
Effects of AEHC on body and feeding behavior in loperamide-induced constipated rats. Bars represent the mean ± SEM. *n* = 12. ^*∗*^*P* < 0.01 versus Group A.

SP rats did not die throughout the course of the experiment, indicating better security and operability of the animal model. Compared with Group A, rats in the model group showed fluffy fur, lassitude, dry stool, and less activity for three days after modeling and the symptoms worsened with the extension of modeling time. The groups treated with medicine have alleviated symptoms as body weight did not differ significantly among all experimental groups, although Group E showed a slightly lower body weight than the other groups (Figure 2(a)). In addition, SD rats with constipation ate significantly less food than Group A (*P* < 0.01), while differences among Groups B, C, D, and E (Figure 2(b)) were not significant. Water consumption also did not change between Group A and Group B. Moreover, no significant increase in water consumption was detected in Groups C, D, and E (Figure 2(c)). Hence, these results show that treatment with the AEHC did not induce any alteration of body weight, food intake, or water consumption.

### 3.3. Effect of AEHC on Stool Number and Moisture Content in Loperamide-Induced Constipation Rats

**Figure 3:**
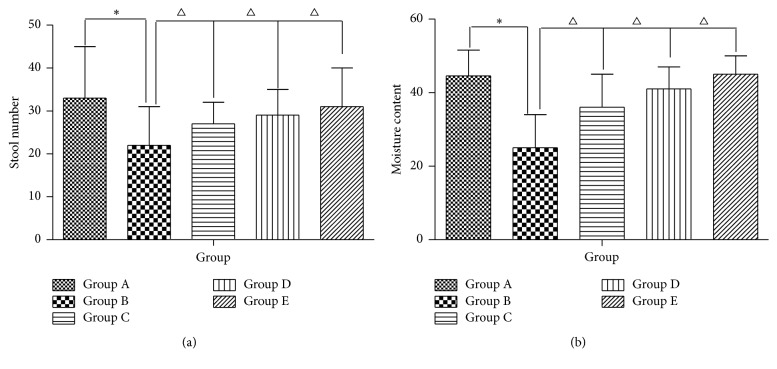
Effects of AEHC on number and moisture content in loperamide-induced constipated rats. Bars represent the mean ± SEM. *n* = 12. ^*∗*^*P* < 0.05 versus Group A; ^△^*P* < 0.05 versus Group B.

Compared with normal feces, the feces in Groups B, C, D, and E were dry, small, and hard and were without burnish before treatment. The stool number and moisture content of feces decreased after all rats were injected with loperamide (*P* < 0.05) but increased when treated by AEHC (*P* < 0.05) (Figures 3(a) and 3(b)).

### 3.4. Effect of AEHC on Small Intestinal Transit Rate in Loperamide-Induced Constipation Rats

**Figure 4:**
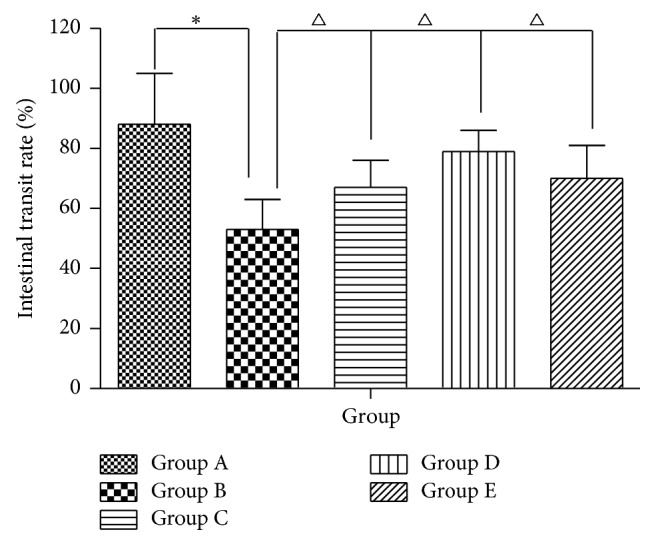
Effect of AEHC on small intestinal transit rate in loperamide-induced constipation rats. Bars represent the mean ± SEM. *n* = 12. ^*∗*^*P* < 0.01 versus Group A; ^△^*P* < 0.05 versus Group B.

As seen from Figure 4, no differences were detected in the baseline small intestinal transit time among the five groups. Group B has a conspicuously lower charcoal powder propelling ratio in comparison with Group A (*P* < 0.01). Although the intestinal transit rate in Groups C, D, and E was also significantly higher than that in Group B (*P* < 0.05), Groups C, D, and E had significantly lower ratios than Group A.

### 3.5. The Intervention with AEHC Partially Enhances Colonic Motility Index Function

**Figure 5:**
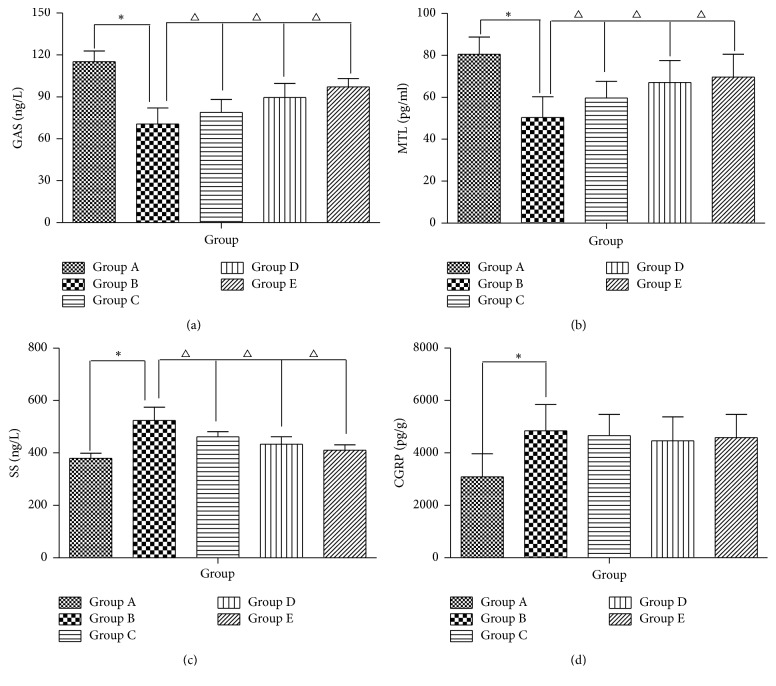
AEHC partially enhances colonic motility index function. Bars represent the mean ± SEM. *n* = 12. ^*∗*^*P* < 0.01 versus Group A; ^△^*P* < 0.05 versus Group B.

The serum levels of GAS and MTL obtained from Group B rats significantly decreased compared with those from Group A (*P* < 0.01, resp.). On the other hand, Groups C, D, and E (*P* < 0.05, resp.) have effectively higher levels of GAS and MTL from the administration of* Herba Cistanche* extracts compared with Group B. The SS values in Group B rats were higher compared with Group A (*P* < 0.01); Groups C, D, and E have attenuated levels of SS (*P* < 0.05) (Figure 5). Compared with constipation rats, the CGRP levels in Group A normal rats (*P* < 0.01) and in* Herba Cistanche*-administered Groups C, D, and E were constant (*P* > 0.05).

### 3.6. Histological Alteration of Colon

**Figure 6:**
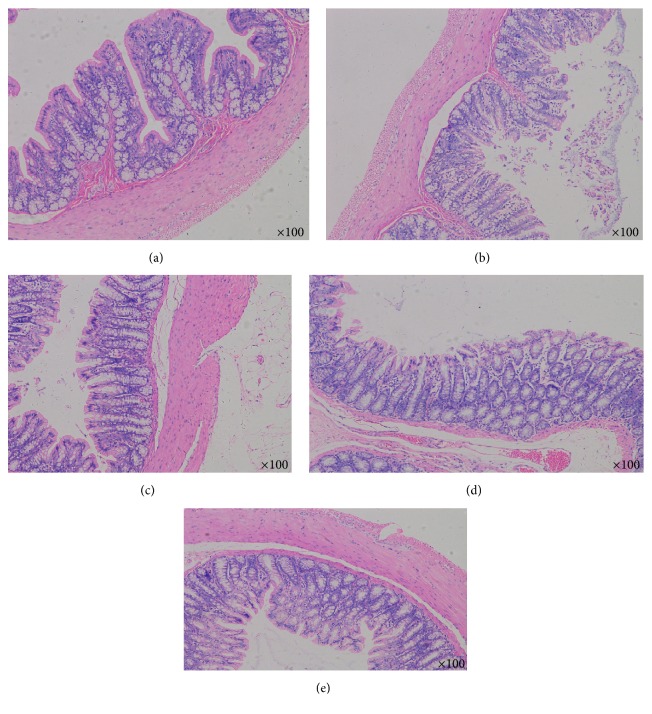
Histological findings in the colon with hematoxylin and eosin staining (*n* = 12, 100x).

**Figure 7:**
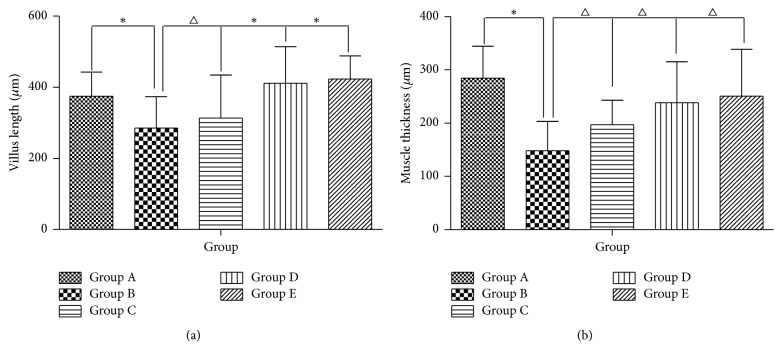
AEHC partially enhances colonic motility index function. Bars represent the mean ± SEM. *n* = 12. ^*∗*^*P* < 0.01 versus Group A; ^△^*P* < 0.05 versus Group B.

To investigate whether AEHC treatment can induce structural alteration of the colon tissue, the villus length, crypt layer thickness, and muscle thickness were measured in transverse colons of rats in the five groups following H&E staining. By comparing the pathology of colon of the rats in every group, the colonic mucosa was smooth and contains small arteries and small veins in the submucosa and a large number of adipose cells. In addition, the muscular layer consisted of smooth muscle cells and the subserosa was complete. The average length of the villus was significantly shorter in Group B than in Group A (*P* < 0.01). However, the average length of the villus in Group C greatly increased by more than 30% when compared with Group B (Figures 6 and 7) (*P* < 0.05). Meanwhile, the average length of the villus greatly increases in Groups D and E when compared with Group B (Figures 6(a) and 6(b)) (*P* < 0.01). Furthermore, alteration of muscle thickness was very similar to villus length. In Group B, the muscle thickness was dramatically thinner when compared with Group A. However, the muscle thickness levels in Groups C, D, and E increased by 30–35% relative to that of Group B (Figures 6 and 7). Overall, these findings indicate that the AEHC may increase the villus length and muscle thickness in the colon of constipated rats.

### 3.7. Administration of AHEC Treatment Increased Protein Expression of c-Kit and SCF

**Figure 8:**
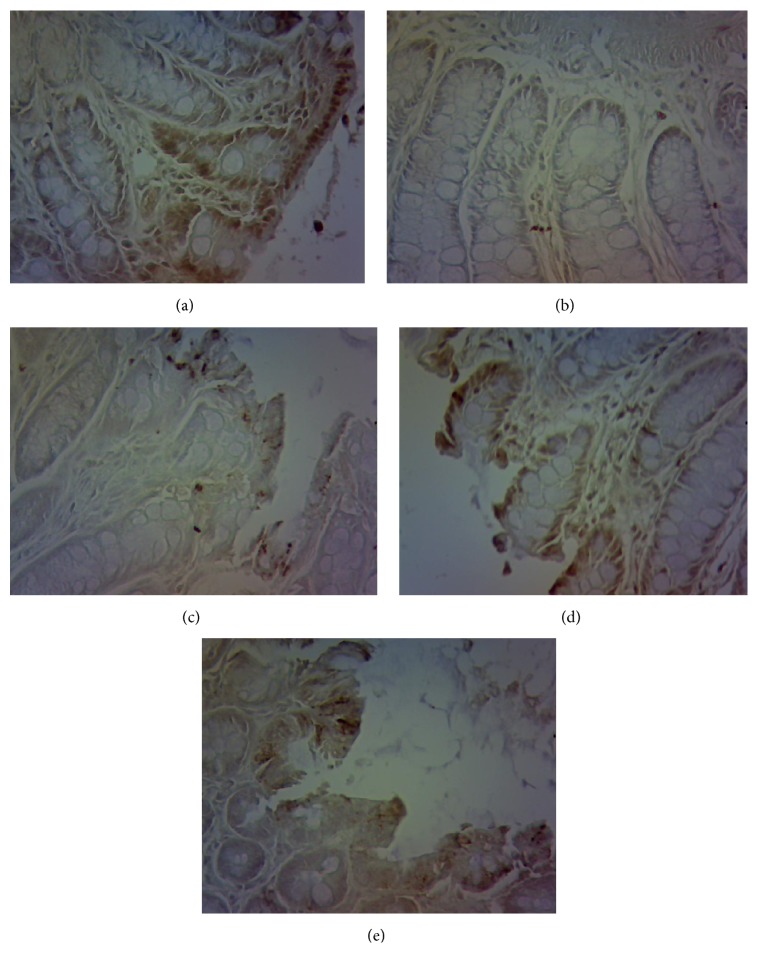
Immunohistochemical staining of the ICC by c-kit (*n* = 12, 200x).

**Figure 9:**
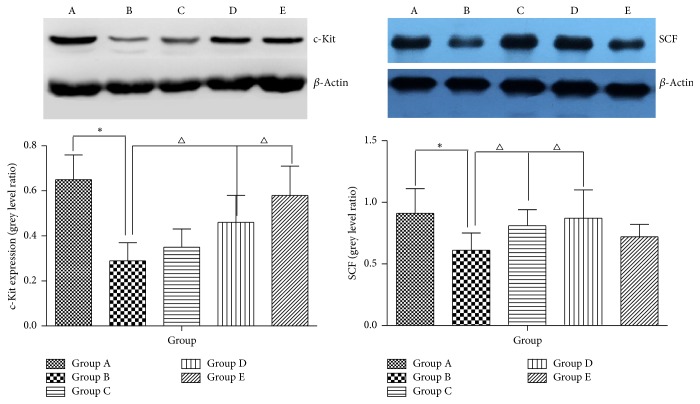
AEHC ameliorated the function of ICC. Data are presented as the mean ± standard deviation. *n* = 8. ^*∗*^*P* < 0.01 versus Group A; ^△^*P* < 0.05 versus Group B.

The immunoreactivity of c-kit was detected in colon as the cells in all groups tested positive for c-kit. As shown in Figure 8, the expression level of c-kit in Group B was lower than that in Group A (*P* < 0.05). The expression level of c-kit in Group C was higher than that in Group B (*P* < 0.05), while more c-kit was present in Groups D and E than in Group B (*P* < 0.05). After treatment with different concentrations of aqueous extract of* Herba Cistanche* for 2 weeks, the protein expression levels of c-kit and SCF were detected by Western blot analysis. Western blot analysis showed that c-kit and SCF expression levels were remarkably lower after loperamide treatment in colons of rats (Figure 9).  Figure 8(e) demonstrates that the decreased c-kit in constipation rats significantly increased by 31.2% after treating the rats with 500 *μ*g/ml AEHC (*P* < 0.05). In addition, treatment with 100 and 200 *μ*g/ml AEHC elevated c-kit protein expression levels. SCF protein expression significantly increased by 20.1, 24.7, and 8.4% at treatment with 100, 200, and 500 *μ*g/ml AEHC, respectively (Figure 9).

### 3.8. Effect of AEHC on mRNA Expression of c-Kit, SCF, and PI3K

**Figure 10:**
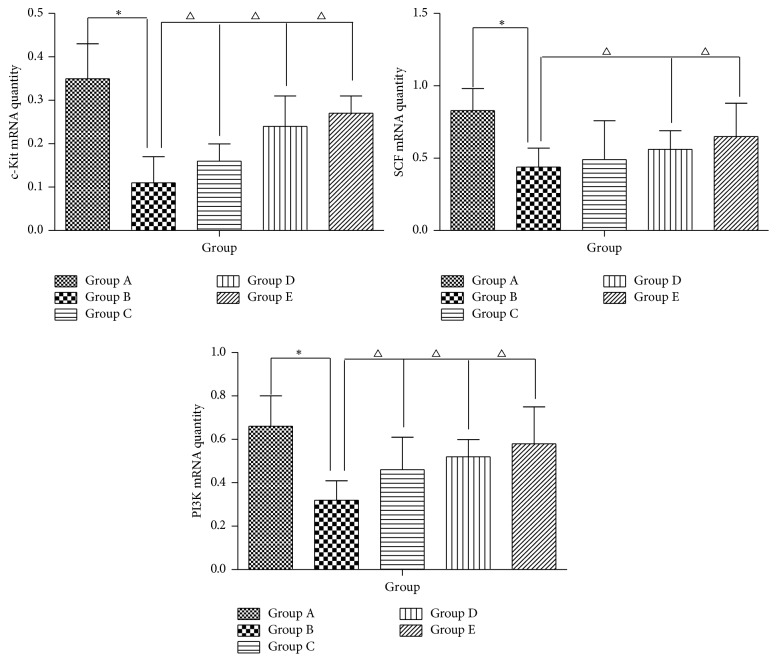
Real-time polymerase chain reaction detection of c-kit, SCF, and PI3K messenger RNA expression. The figure shows the representative result from repeated experiments (*n* = 8). Data are expressed as mean ± standard deviation. Compared with Group A, ^*∗*^*P* < 0.01; compared with Group B, ^△^*P* < 0.05.

To investigate whether AEHC treatment can affect the regulation of mRNA related to muscle contraction, alterations in c-kit, SCF, and PI3K expression levels were observed in colons of constipation rats using specific primers. The results showed that the mRNA levels of c-kit in Group B was significantly lower compared with Group A (*P* < 0.05), while the mRNA levels in Groups C, D, and E were higher than in Group B (*P* < 0.05) (Figure 10). We also examined the mRNA expression of SCF, a ligand of c-kit. The mRNA expression of SCF in Group B rats was significantly lower than in Group A, whereas treatment with AEHC in Groups C, D, and E increased SCF mRNA expression (Figure 10). In addition, we examined the effect of AEHC on PI3K gene expression. On day 14, PI3K gene expression level was significantly higher in Groups C, D, and E than in Group B (*P* < 0.05).

## 4. Discussion

Constipation is a highly common disorder characterized by poor bowel movements. It greatly diminishes quality of life and brings about huge economic burdens on both patients and national health insurance [32]. Natural product and medicinal food now attract ever-increasing attention because of their potential to become new drugs used for treatment of constipation [37, 38]. Therefore, we studied the therapeutic effects of the AEHC on loperamide-induced constipated rats.

Loperamide, atropine-diphenoxylate, and morphine are widely used to induce constipation of laboratory animals. Among the three drugs, loperamide induces prolonged duration of stool evacuation and a delay of intestinal luminal transit, since it inhibits both water secretion and smooth movement in the intestinal wall [39].

Analysis of body weight in this study did not find any difference within each group. After the experiment, no difference was found either. The low-dosage group of AEHC still saw the lowest body weight, yet the model group and the normal group witnessed the highest body weight. Constipation is generally a functional disease. Slow transit constipation induced in this study did not affect nutrient uptake of the rats and their body weight during the experiment. Difference in amounts of feces of the rats and their moisture content did not exist between groups before modeling; however, they both decreased remarkably after modeling and the moisture content of feces increased after using AEHC at different dosages. After studying the propellant rate of carbon powder of colon of rats, the model group saw obvious decrease of the rate, while it remained normal in low-dosage, medium-dosage, and high-dosage groups of AEHC. The results showed that loperamide-induced chronic transit constipation treated with different dosage of* Herba Cistanche* can recover the propellant function of colon to different degrees.

Gastrointestinal hormones are high-performance bioactive substances secreted by endocrine cells of gastrointestinal mucosa and pancreas, possessing effects of both excitement and inhibition to regulate gastrointestinal motor function [40, 41], of which GAS, MTL, and SS have major effect. The former two hormones stimulate secretion of digestive juice, constrict gastrointestinal smooth muscle, promote the movement of gastrointestinal contents, and excite the gastrointestinal motility. SS, on the contrary, inhibits the secretion of digestive juice, contraction of gastrointestinal smooth muscle, and gastrointestinal emptying [42]. GAS and MTL levels in the colon tissue of the model group were found to be the lowest, indicating that the modeling method certainly lowered the concentration of GAS and MTL in colon tissue. As the main gastrointestinal hormones of colonic motility, the concentration of GAS and MTL increased by different dosages of AEHC and thus enhanced colonic motility. In contrast, SS level in the colon tissue of the model group was the highest, indicating that the modeling method can promote the concentration of SS in colon tissue. CGRP is one of the most powerful vasodilators and its relaxation effect is 10-fold stronger than contraction effect. As an important neurotransmitter or neuromodulator that regulates digestive functions, CGRP can inhibit the majority of gastrointestinal motility and the secretion of various digestive juices [43]. The concentration of CGRP in the model group was higher than Group A, indicating that the modeling method can increase CGRP concentration in the colon tissue, which may also be one of the factors for reduced colonic peristalsis which will result in constipation. CGRP concentration in the colon tissue of AEHC groups was lower than that in the model group, suggesting that AEHC had relatively weak inhibitory effect on colon movement.

c-Kit is a transmembrane protein. The stem cell factor (SCF) of its ligand is produced by neuron cells and smooth muscle cells. It promotes the development and differentiation of ICC and maintains its normal physiological function. c-Kit labeling indirectly reflects the quantity and density of ICC [44, 45]. In addition, ICC cell plays a key role in regulating gastrointestinal motility and acts as the pacemaker of gastrointestinal movement. So, some disorders of gastrointestinal motility may find a decline of the quantity and function of ICC cells. According to previous studies, cell counts in the colon of patients with chronic transit constipation were reduced. Hence, the present study observed the colonic cells of rats by using specific expression and immunohistochemical technique. Compared with normal group, the quantity of cells in the model group decreased remarkably but increased after treatment. The study showed that ICC cells and stem cell factors (SCF) are closely related to c-kit/SCF signal pathway. SCF is the natural ligand of c-kit expressed in various tissues of the body but mainly produced by the stromal cells in the bone marrow. Every c-kit monomer combines with a SCF through the extracellular domains 1–3. After SCF dimerization, the structure of c-kit monomer changes and produces homodimerization, which then results in the autophosphorylation of amino acid residue in the cell membrane and stimulates various second signal molecules to regulate the cellular functions of ICC. The second signal molecule is phosphatidylinositol 3-kinase (PI3K), an apolipoprotein that converts 4,5-diphosphoinositide into 3,4,5-triphosphoinositide through the combination with tyrosine 721 of c-kit. Previous research showed that PI3K inhibitors Wortmannin and LY294002 gave rise to abnormal development of ICC by blocking the signal [46]. Our experiment found that c-kit and SCF protein and mRNA expressions in the model group decreased at first but then increased in AEHC groups of different dosages, albeit at below-normal levels. mRNA expression of PI3K increased remarkably. Therefore, SCF/c-kit may take effect through PI3K. The results showed that AEHC could regulate the smooth muscle of gastrointestinal tract by increasing the quantity of ICC cells. Low dosage of AEHC had no significant effect on constipation, while its medium dosage and high dosage shortened the first defecation time of the rats, increased moisture content of the feces, improved propellant rate of the colon, refined the quantity of the feces, increased GAS, MTL, and CCK levels, and strengthened the colonic contractility. The effective dosage of AEHC is initially 20 g/d after conversion, which is equivalent to the medium dosage and high dosage in this study.

## 5. Conclusions

AEHC promotes intestinal motility by improving ICC function and regulating neurotransmitters in this study, which proved that AEHC has the potential to treat and prevent constipation. The extracts improved the slow-wave production of colon and regulated the rhythm of the contractive activity of smooth muscle by increasing ICC quantity through the PI3K/SCF/c-kit signal pathway.

However, further more detailed experimental studies might improve understanding of the other molecular pathways of AEHC in curing STC and help guide prospective clinical studies evaluating its effects and safety.

## Figures and Tables

**Table 1:** Targeted genes and internal parameter of primer.

Amplified products	Sizes	Sequence
c-Kit	212 bp	Downstream: 5′-CTGGCTGCCAA ATCTCTGTGAA-3′
		Upstream: 5′-AGATGACGAGCTGGCTCTGGA-3′
SCF	119 bp	Downstream: 5′-GGAGATGGCAGTTGTGACTA-3′
		Upstream: 5′-CATGCTTTAAGGCCTTTGTCACGA-3′
PI3K	159 bp	Downstream: 5′-TGGGCACAGGGAAGACAA-3′
		Upstream: 5′-ACCAGTTGGCTCGGCATA-3′
GAPDH	252 bp	Downstream: 5′-CAGGATTCCATACCCAAG-3′
		Upstream: 5′-ACACTGTGCCCATCTACG-3′
